# Interaction between Long Noncoding RNAs and Syncytin-1/Syncytin-2 Genes and Transcripts: How Noncoding RNAs May Affect Pregnancy in Patients with Systemic Lupus Erythematosus

**DOI:** 10.3390/ijms24032259

**Published:** 2023-01-23

**Authors:** Rossella Talotta

**Affiliations:** Rheumatology Unit, Department of Clinical and Experimental Medicine, University of Messina, AOU “G. Martino”, via Consolare Valeria 1, 98124 Messina (ME), Italy; talotta1@virgilio.it or rtalotta@unime.it

**Keywords:** bioinformatics, epigenetics, human endogenous retroviruses, long noncoding RNAs, placenta, pregnancy, syncytin-1, syncytin-2, syncytiotrophoblast, systemic lupus erythematosus

## Abstract

Background: Patients with systemic lupus erythematosus (SLE) often suffer from obstetric complications not necessarily associated with the antiphospholipid syndrome. These events may potentially result from the reduced placental synthesis of the fusogenic proteins syncytin-1 and syncytin-2, observed in women with pregnancy-related disorders. SLE patients have an aberrant noncoding (nc)RNA signature that may in turn dysregulate the expression of syncytin-1 and syncytin-2 during placentation. The aim of this research is to computationally evaluate and characterize the interaction between syncytin-1 and syncytin-2 genes and human ncRNAs and to discuss the potential implications for SLE pregnancy adverse outcomes. Methods: The FASTA sequences of the syncytin-1 and syncytin-2 genes were used as inputs to the Ensembl.org library to find any alignments with human ncRNA genes and their transcripts, which were characterized for their tissue expression, regulatory activity on adjacent genes, biological pathways, and potential association with human disease. Results: BLASTN analysis revealed a total of 100 hits with human long ncRNAs (lncRNAs) for the syncytin-1 and syncytin-2 genes, with median alignment scores of 151 and 66.7, respectively. Only lncRNAs TP53TG1, TTTY14, and ENSG00000273328 were reported to be expressed in placental tissue. Dysregulated expression of lncRNAs TP53TG1, LINC01239, and LINC01320 found in this analysis has previously been described in SLE patients as well as in women with a high-risk pregnancy. In addition, some of the genes adjacent to lncRNAs aligned with syncytin-1 or syncytin-2 in a regulatory region might increase the risk of pregnancy complications or SLE. Conclusions: This is the first computational study showing alignments between syncytin-1 and syncytin-2 genes and human lncRNAs. Whether this mechanism affects syncytiotrophoblast morphogenesis in SLE females is unknown and requires further investigation.

## 1. Introduction

Systemic lupus erythematosus (SLE) is a prototypical autoimmune connective tissue disease that mainly affects women of childbearing age. The worldwide prevalence is reported to range from 36.7 to 366.6 per 100,000 individuals, with various differences according to gender and ethnicity [1]. The pathogenesis is multifactorial: genetics and environmental factors such as ultraviolet light and infections can trigger the disease by chronically activating both innate and adaptive immune responses. The immunological scenario is based on the activation of dendritic cells, neutrophil granulocytes, B and T lymphocytes, and the complement system, leading to mechanisms such as NETosis, autoantibody release, immune complex formation, and type I interferon (IFN) production [2]. SLE is a multiorgan disease with both constitutional and organ-specific manifestations. The latter include renal, hematological, neuropsychiatric, mucocutaneous, musculoskeletal, and serosal symptoms [3]. According to a meta-analysis conducted in the United States with more than 26,000 patients, the overall mortality risk is almost threefold-increased in SLE compared to the general population [4]. The main causes of death are attributed to renal failure, infections, and heart disease [1]. In women of childbearing age, SLE is commonly associated with obstetric complications such as fetal growth restriction, spontaneous abortion, preeclampsia, cesarean section, and preterm delivery. These events can occur in 12% to nearly 40% of SLE women and are dependent on age, disease activity, concomitant medications, autoantibody titers, including antiphospholipid antibodies (aPLs), and type I IFN activity [5]. Underlying mechanisms may encompass both local vasculopathy and inflammation due to trophoblastic antigen recognition. In particular, positivity of aPLs is a risk factor for antiphospholipid syndrome (APS), an autoimmune disorder characterized by pregnancy morbidity and recurrent venous and/or arterial thrombosis. It is estimated that approximately 40% of SLE patients have circulating aPLs, although APS occurs in less than 40% of aPL-positive SLE patients [6]. According to one study, aPL-negative SLE women have twice the risk of obstetric complications such as perinatal death and preterm delivery compared with the general population, suggesting the existence of additional pathogenic pathways [7].

Placental development is controlled by several mediators, two of which are represented by the fusogenic glycoproteins syncytin-1 and syncytin-2. As the name suggests, the main role of syncytins is to induce the fusion of single nucleated cytotrophoblast cells into syncytiotrophoblast, which divides maternal and fetal tissues and has a crucial function in fetal protection and nutrition [8]. Both syncytin-1 and syncytin-2 are involved in the homeostasis, differentiation, proliferation, and survival of syncytiotrophoblast and have additional immunomodulatory properties that induce maternofetal tolerance thanks to the immunosuppressive domain (ISD), which counteracts the activation of dendritic cells, T cells, and the production of IFNs [9,10,11]. Syncytin-1 and syncytin-2 are endogenous retroviral proteins encoded by the envelope gene of proviruses belonging to the human endogenous retrovirus (HERV)-W and HERV-FRD families, respectively. HERVs are genetic elements of ancient retroviral infectious origin that constitute approximately 8% of the human genome. After their integration into the nuclear genome, HERVs have been increasingly silenced by inactivating mutations, although some of them may still retain intact open reading frames (ORFs) and consequently reactivate under physiological or pathogenic circumstances [12]. The expression of syncytin-1 and syncytin-2 transcripts and proteins during pregnancy is a fair example of physiological HERV reactivation. This process is tightly regulated by epigenetic mechanisms and is critical for pregnancy outcome.

On the other hand, abnormal reactivation of HERVs has been linked to the occurrence of human diseases, including autoimmune disorders [13]. In SLE patients, dysregulation of HERV-E members has been found to mimic viral infection and further stimulate IFN response and the production of antibodies against nuclear components [14]. This event may be embedded in a more complex scenario involving other noncoding (nc)RNAs that provide epigenetic and posttranscriptional control of coding and noncoding genes. Studies have shown that SLE is associated with a dysregulated signature of microRNAs (miRNAs) and long ncRNAs (lncRNAs) in peripheral blood mononuclear cells (PBMCs) and kidney tissue [15]. It follows that the alteration of the SLE transcriptome profile could be the basis for impaired expression of syncytin-1 and syncytin-2 during pregnancy, providing an alternative pathogenic view to explain obstetric complications in these patients.

The aim of this research is to computationally evaluate and characterize the interaction between syncytin-1 and syncytin-2 genes and human ncRNAs and to discuss the potential implications for SLE pregnancy adverse outcomes.

## 2. Results

### 2.1. Syncytin-1 BLASTN Analysis against Human ncRNA Genes

BLASTN analysis against human ncRNA genes on the Ensembl.org database yielded 100 hits, all of which were lncRNAs, Table S1.

The alignment score ranged between 63.8 and 1650 (median value 151; IQR 344); the percentage of identity was between 79.3% and 97.4% (mean ± SD 89.3 ± 3.7%) and nucleotide length ranged between 36 and 1312 (median value 132; IQR 309.2).

According to the GeneCards database, retrieved lncRNAs appeared to be mainly expressed in the gonads and reproductive tissue, followed by gastrointestinal apparatus and the exocrine glands. Interestingly, placental expression was only reported for two lncRNAs (TP53TG1 and TTTY14) under physiological circumstances. The retrieved lncRNAs WARS2-AS1, LINC01239, LINC00383 and TP53TG1 were found to be associated with the risk of certain types of cancer or genetic diseases, while no associations between other lncRNAs and human disease were found.

Enrichment analysis showed that retrieved lncRNAs are mostly involved in platinum drug resistance, nonalcoholic fatty liver disease, and the cell cycle, Figure S1.

Twenty-six hits fell in DNA regulatory regions (15 enhancers, 1 CCCTC-binding factor or CTCF, 7 promoters, 2 promoters + promoter flanks, 1 promoter + promoter flank + enhancer + CTCF). Adjacent coding genes located within 0.5 Mb backward and forward of the lncRNA genes and associated disorders are listed in Table 1.

By using the bioinformatic tool QmRLFS-finder, there was a single case in which the nucleotide sequence of a lncRNA complementary to syncytin-1 gene was predicted to form an R-loop. The involved lncRNA was SLC17A6-DT, normally expressed in the brain, muscle, gastrointestinal tract, glands, and testis.

RNAct tool predicted a total of 27 interactions with RNA-binding proteins (RBPs) (12 with nischarin or NISCH; 12 with AE-binding protein 2 or AEBP2; 2 with transcription elongation factor SPT5 or SUPT5H; 1 with cysteine-rich hydrophobic domain-containing protein 1 or CHIC1), Table 2. The mean ± SD prediction score ranged from 24.4 ± 6.7 for NISCH to 21.8 ± 5.6 for AEBP2, 18.28 for CHIC1, and 17.65 for SUPT5H.

### 2.2. Syncytin-2 BLASTN Analysis against Human ncRNA Genes

Similar to syncytin-1, a total of 100 alignments were found between syncytin-2 gene and human lncRNAs, Table S2.

The alignment score ranged from 51.9 to 346 (median value 66.7; IQR 53.6); the percentage of identity was between 78.5% and 95.6% (mean ± SD 84.6 ± 3.7%) and the nucleotide length ranged between 63 and 985 (median value 100; IQR 107).

By consulting the GeneCards database, the retrieved lncRNAs appeared to be mostly expressed in the testis and nervous system. Under physiological circumstances, the placental expression was solely reported for the lncRNAs ENSG00000273328 and TP53TG1.

Enrichment analysis showed that the retrieved lncRNAs are mainly involved in cardiac muscle contraction and adenosine monophosphate (AMP)-activated protein kinase (AMPK) pathways, Figure S2. With the exception of the lncRNAs SCAT1, LINC00320, PURPL, and TP53TG1, associated with the risk of certain types of cancer or genetic diseases, no other associations with human disorders were found.

Thirty hits fell in lncRNA regulatory regions (18 enhancers, 10 CTCFs, 1 CTCF + promoter, and 1 promoter). Adjacent coding genes (within 1 Mb) and related diseases are listed in Table 3.

No R-loops were predicted for the syncytin-2-aligned sequences of retrieved lncRNAs.

RNAct predicted a total of 24 interactions between retrieved lncRNA transcripts and RBPs (11 with NISCH; 6 with AEBP2; 6 with CHIC1; and 1 with DNAJ heat shock protein family (Hsp40) member C5 beta or DNAJC5B), Table 4. The mean ± SD prediction scores ranged from 21.2 ± 8.2 for AEBP2 to 20.1 ± 5.8 for NISCH, 15.08 ± 5.2 for CHIC1, and 16.73 for DNAJC5B.

## 3. Discussion

The results of this pivotal in silico study show that syncytin-1 and syncytin-2 genes and transcripts are at the center of an intricate epigenetic network involving coding genes and lncRNAs. The latter are ncRNAs with more than 200 nucleotides that have recently attracted the attention of researchers due to their pathogenic potential in diseases such as cancer, neurodegenerative disorders, and autoimmunity [16]. Dysregulation in the lncRNA signature may also be responsible for impairing critical trophoblast cell functions such as proliferation, migration, invasion, and cell cycle progression [17]. More than 50,000 lncRNAs have been discovered in intergenic or intron/exon regions of coding genes of the human genome, but most have not yet been characterized in terms of their biological functions [18]. LncRNAs have been localized to both the nucleus and cytosol. Nuclear lncRNAs play a crucial role in scaffolding and remodeling chromatin and regulating transcription by binding RBPs and DNA and generating R-loops, which are trimeric DNA-RNA hybrids [19]. On the other hand, cytosolic lncRNAs could control the translation and stability of proteins as they can bind to RBPs, mRNAs, or miRNAs [18].

It has been postulated that lncRNAs could override other noncoding transposable elements, including long interspersed nuclear elements (LINEs), short interspersed nuclear elements (SINEs), and HERVs. In turn, HERV-derived solitary long terminal repeats (LTRs) may provide regulatory sequences and control the expression of neighboring lncRNA genes [20].

**Figure 1:**
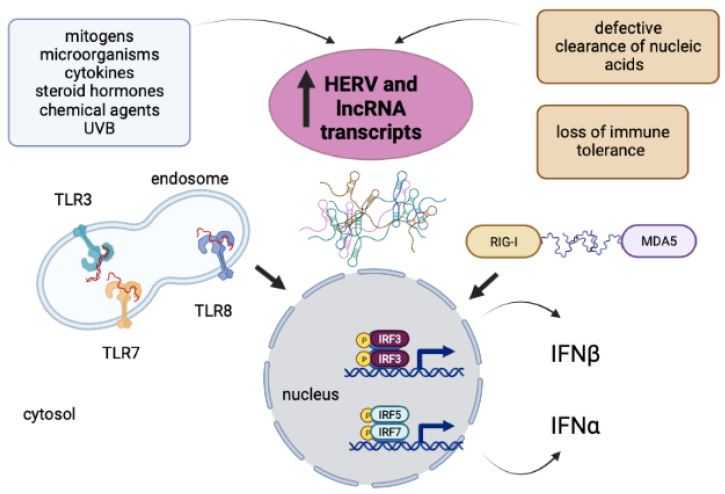
Cascade of events triggered by ncRNAs leading to type I IFN response in SLE. Under the stimulus of mitogens, microorganisms, proinflammatory cytokines, hormones, UVB, or chemical agents, the cells of SLE patients may overproduce ncRNA transcripts. Due to defective clearance of nucleic acids and the loss of immune tolerance that characterize SLE, ncRNAs may bind to RNA sensors such as TLRs, RIG-I, and MDA5 and contribute to the development of the type I IFN response and autoimmunity (HERV: human endogenous retrovirus; IFN: interferon; IRF: interferon regulatory factor; lncRNA: long noncoding RNA; MDA5: melanoma differentiation-associated gene 5; RIG-I: retinoic acid-inducible gene I; TLR: Toll-like receptor; UVB: ultraviolet B radiation). The figure was created with BioRender.com.

SLE patients exhibit a dysregulated ncRNA signature that results in the increased expression of transcripts and antigenic proteins derived from HERV members or lncRNAs [14,15,21,22,23,24,25,26]. This event may depend on the SLE cytokine milieu, hormones, chemicals or microbial stimuli [27,28]. The overproduction of nucleic acids may foment the type I IFN response and the release of anti-dsDNA antibodies, both of which have been associated with SLE obstetric complications [28,29,30], Figure 1.

In addition, lncRNA transcripts may trigger an epigenetic mechanism to control the expression of other coding and noncoding genes, some of which, such as HERVs, may play important roles in certain life stages such as pregnancy. As mentioned previously, SLE patients exhibit aberrant HERV expression compared to healthy controls [14,31], which in turn may reflect an altered lncRNA transcriptome. Unfortunately, there are no studies investigating the occurrence of dysregulated synthesis of the HERV-derived env proteins syncytin-1 and syncytin-2 in SLE patients. The role of syncytins in APS pathogenesis also remains unknown, although some recent studies have reported an altered lncRNA signature in these patients compared with controls [32,33]. Indeed, lncRNAs may contribute to several steps of APS pathogenesis, including leukocyte activation, immunothrombosis, and impaired embryonic development [34], but whether these events are influenced by the abnormal expression of syncytin genes has not been investigated to date.

The transmembrane glycoprotein syncytin-1 is encoded by the HERV-W provirus ERVWE1 at the 7q21.2 env locus, whereas syncytin-2, which is homologous to syncytin-1, is encoded by an HERV-FRD provirus at locus 6p24.1 [35]. The upregulation of syncytin-1 in villous and extravillous trophoblasts depends on the binding of the transcription factors cAMP-response element-binding protein (CREB), glial cells missing transcription factor 1 (GCM1), and the hypomethylation of a neighboring MaLR solitary LTR. Expression of syncytin-2 is instead restricted to the villous cytotrophoblast and regulated by GCM1 binding and methylation patterns [35]. Therefore, syncytin-1 and syncytin-2 exhibit distinct cellular expression patterns and time-dependent effects as they separately regulate cell cycle phases in trophoblast cells [36]. Normally, the expression of syncytins is directly proportional to gestational age, and a decrease has been associated with pathological conditions such as hypoxia and preeclampsia [37,38,39]. However, it is unknown whether the differential expression of syncytin genes during placentation could be under the epigenetic control of lncRNAs.

Moreover, according to the results of a preclinical study, syncytin-2, but not syncytin-1, might have immunosuppressive effects through its ISD [40]. These results are consistent with the immunopathogenic activity of HERV-W env proteins, which can stimulate both innate and adaptive immunity [35]. The immunogenicity of HERV-W env proteins seems to be most prominent in neuroinflammatory diseases such as multiple sclerosis (MS) [28], while data concerning SLE pathogenesis are still unclear [41,42].

In this analysis, a total of 100 human lncRNA transcripts were predicted to align with the nucleotide sequence of syncytin-1 or syncytin-2. An aberrant lncRNA transcriptome in the endometrium and placental tissue has been described in association with obstetric complications in women without SLE [17,43,44,45,46,47,48]. When comparing such literature data with the results of this study, a match was found only for the lncRNAs TP53TG1, LINC01320, and LINC00320 [45,46,47,48]. Other lncRNAs retrieved in the present analysis have been associated with an increased risk of endometriosis or X chromosome instability during early embryonic development [49,50,51,52,53]. Among them, the lncRNA TP53TG1 was predicted to align with the nucleotide sequences of both syncytin-1 and syncytin-2 with scores of 140 (ID: 89.4%) and 51.9 (ID: 92.1%), respectively. TP53TG1 can be localized both intracellularly (nuclear and cytosolic localization) and extracellularly in placental tissue and appears to be involved in cell damage that can result from exposure to agents such as ultraviolet radiation [54], which is a crucial triggering factor for SLE. Although the actual role of TP53TG1 in pregnancy is unknown, one study found increased demethylation of this lncRNA gene in the female cadmium-exposed placenta, which may be responsible for suboptimal fetal growth [45].

Indeed, SLE females might have a different transcriptomic signature than non-SLE females with pregnancy adverse outcomes. However, only a few of the lncRNAs found in this computational analysis have been previously reported in the literature as biomarker candidates for SLE risk [55,56,57,58], Table 5. Interestingly, a recent experimental study characterizing the molecular signature of 54 biopsy specimens from lupus nephritis patients demonstrated that the aforementioned lncRNA TP53TG1 inversely correlated with the degree of glomerulosclerosis [55]. Conversely, none of the lncRNAs reported in studies of APS patients correlated with lncRNAs that showed alignment with syncytin-1 or syncytin-2 genes [32,33].

These discrepancies may be due to the different methodologies and selective tissue expressions of lncRNAs. With only one exception, the studies that aimed to characterize the lncRNA transcriptome in SLE patients did not include pregnant women or analyze placental tissue. The lncRNA profile in the placenta of SLE women was characterized only in a recent Chinese RNA-seq study [59]. Samples were collected between 34.9 and 39.7 weeks of gestation; in 10% of cases, fetal weight was below the 10th percentile, and in two cases, the patients had a cesarean section. The results showed a total of 52 dysregulated lncRNAs in the placental tissue of SLE women compared with controls. Again, none of the 52 lncRNAs reported by the authors correlated with the lncRNAs found in the present study. Different methods, the small sample (10 participating SLE patients), the low SLE disease activity, and the absence of severe obstetric complications such as preeclampsia or fetal loss may be the reasons for these conflicting results.

Although tissue expression was not always available, a number of lncRNAs complementary to syncytin-1 and syncytin-2 have been reported to be physiologically expressed in placental or uterine tissue. In the GeneCards database, placental localization has been described for the lncRNAs TP53TG1, TTTY14, and ENSG00000273328. Two of them (TP53TG1 and TTTY14) have been reported to be associated with pregnancy adverse outcomes or gynecological diseases. In addition to the previously mentioned TP53TG1 [45], the Y-linked lncRNA TTTY14, which aligns with the syncytin-1 sequence, is localized in the nucleus and may be abnormally expressed in the endometrium as a phenomenon of male microchimerism in endometriosis and infertility [53]. Therefore, overexpression of TP53TG1 and TTTY14 in the placenta or uterus could be responsible for pregnancy complications. As suggested by the present study, a hypothetical underlying mechanism could be complementation and/or sequestration of syncytin-1 and syncytin-2 genes or transcripts, ultimately leading to inhibition of their translation into functional glycoproteins.

The results of the enrichment analysis for the detected lncRNA genes showed different Kyoto Encyclopedia of Genes and Genomes (KEGG) pathways depending on the type of matching syncytin sequence. Specifically, lncRNA genes that had hits with the syncytin-2 sequence were predicted to be involved in the AMPK signaling pathway, which regulates cellular energy homeostasis. AMPK function has been shown to be required during placental differentiation, providing nutrient transport and protection of both maternal and fetal tissues and, consequently, preventing preeclampsia, intrauterine growth restriction, and preterm birth [60]. Conversely, the results of enrichment analysis for lncRNA genes matching the syncytin-1 sequence showed an association with metabolic pathways occurring in the liver leading to nonalcoholic fatty liver disease, which in turn is associated with pregnancy complications [61,62]. In neither case did the KEGG pathways converge with a dysregulated immune response or alterations in autophagy or phagocytosis, which are hallmarks of SLE pathogenesis [63,64].

Because lncRNAs can epigenetically regulate transcription of neighboring genes, chromosomal regions near (within 1.00 Mb) lncRNA genes that contained hits for syncytin-1 and syncytin-2 genes in a regulatory region were analyzed. Protein-coding genes located near syncytin-1-matched lncRNA genes were found to be involved in DNA binding and transcription, stress response, anabolic or metabolic pathways, enzyme reactions, and exocrine gland secretion. Among them, the *CNR1* and *GRIN2B* genes, encoding cannabinoid receptor 1 and NMDA-type ionotropic glutamate receptor subunit 2B, respectively, have been associated with the risk of preeclampsia in case-control studies [65,66]. Importantly, a novel association between SLE and the *GRIN2B* gene was found in a dataset from a genome-wide association study (GWAS) [67].

Instead, genes bordering syncytin-2-matched lncRNAs were found to be involved in metabolism, cell motility, transcription, translation, ubiquitination, immune defense, and nociception. Polymorphic variants of *CYLD*, *GC*, *MBL2*, and *ZNF572* genes have been collectively associated with the risk of preterm birth or recurrent late pregnancy loss [68,69,70,71,72], while dysregulated expression of *CYP8B1* may be responsible for pregnancy intrahepatic cholestasis in mice [73]. The *TCL6* gene has been reported to be overexpressed in the placental tissue of women with preeclampsia, threatened miscarriage, or spontaneous abortion [74,75]. Finally, the neuropeptide receptor FF 2, encoded by the gene *NPFFR2*, was found to be overexpressed in the placental tissue of women with preeclampsia and closely related to the production of syncytin-1 and syncytin-2 during pregnancy [36]. Importantly, there is evidence that some of the genes listed above may be critical for the pathogenesis of SLE. These genes include *CYLD* and *MBL2*, both of which are involved in the innate immune response. *CYLD*, encoding a deubiquitinase, appears to be overexpressed in kidney samples from patients with SLE glomerulonephritis [76]. On the other hand, more than 30 publications report an association between polymorphic variants of the *MBL2* gene, encoding mannose-binding lectin 2, and SLE [77,78]. Mannose-binding lectin activates complement and is thus involved in the clearance of cellular debris and pathogens [79]. Low levels of mannose-binding lectin have been associated with the risk of disease. Indeed, a deficit in its function could be crucial for the loss of immune tolerance and the development of autoimmune phenomena. Associations between protein-coding genes adjacent to lncRNAs of interest and SLE or pregnancy complications are shown in Table 6.

This analysis also showed that the lncRNA SLC17A6-DT contains an alignment to syncytin-1 in an R-loop-forming sequence, but the pathogenic role of this lncRNA in autoimmunity or pregnancy complications remains to be elucidated.

Finally, a total of 27 and 24 interactions with RBPs were predicted for lncRNAs aligned with syncytin-1 and syncytin-2, respectively. The RBPs included NISCH, AEBP2, SUPT5H, CHIC1, and DNAJC5B, which preside over various processes including cell proliferation and malignant transformation, neural crest migration, transcription elongation, and antiviral defense [80,81,82,83,84]. Interestingly, CHIC1, predicted to bind one syncytin-1-complementary lncRNA and six syncytin-2-complementary lncRNAs, was reported to be progressively hypermethylated and consequently hypo-expressed from morula to blastocyst development in an animal experiment under physiological conditions [85]. However, the relationship between CHIC1 and the expression of syncytins during pregnancy in humans has not yet been investigated.

**Figure 2:**
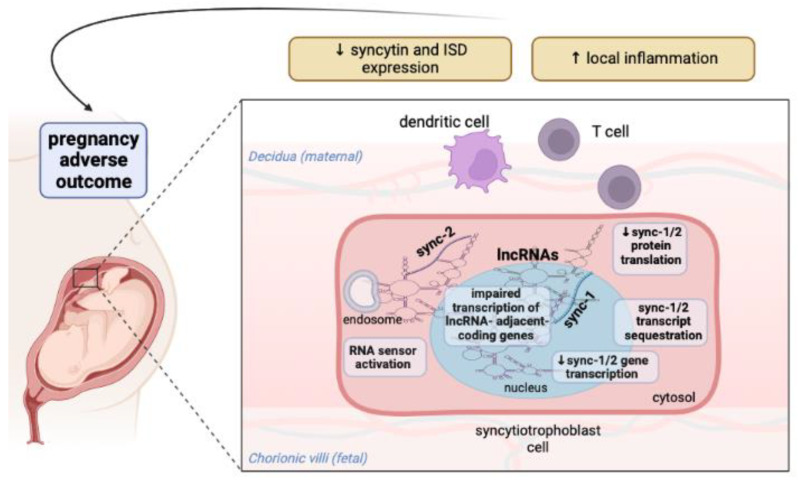
Hypothetical mechanisms triggered by abnormal expression of lncRNAs in the syncytiotrophoblast of pregnant women with SLE. LncRNAs may associate with syncytin-1 and syncytin-2 genes or transcripts in the nucleus or cytosol. Such interactions may prevent transcription of syncytin-1 and syncytin-2 genes, induce sequestration of transcripts, or affect mRNA splicing and translation. Meanwhile, nucleic acid accumulation in the cytosol could fuel inflammation by stimulating RNA sensing platforms and triggering a type I IFN response. The result would be decreased expression of syncytin-1 and syncytin-2 and local inflammation with adverse effects on pregnancy outcome. On the other hand, alignment of lncRNAs with the syncytin-1 or syncytin-2 nucleotide sequence may affect transcription of neighboring protein-coding genes involved in placentation or the control of inflammation (ISD: immunosuppressive domain; lncRNAs: long noncoding RNAs; sync: syncytin). The figure was created with BioRender.com.

In summary, this computational study shows that the genes and transcripts of syncytin-1 and syncytin-2 correspond to human lncRNAs, which have both nuclear and cytosolic localization and may be involved in energetic and metabolic pathways. Remarkably, three of the lncRNAs found (TP53TG1, LINC01239, and LINC01320) have been described in studies to be dysregulated in both SLE patients and women with high-risk pregnancies. In addition, protein-coding genes adjacent to the lncRNAs found have been reported to be associated with gynecologic/obstetric complications in nine cases and with SLE risk in three cases. Although no data are currently available to confirm these pivotal findings, the following hypothesis can be made. Abnormal nuclear and cytoplasmic expression of lncRNAs in the syncytiotrophoblast and placental tissue of pregnant women with SLE might prevent the transcription and translation of syncytin-1 and syncytin-2 mRNA. Moreover, the spongy effect of complementary lncRNAs could be thought to locally reduce maternal immune tolerance to embryonic tissues by sequestering syncytin transcripts and preventing surface expression of the ISD. Increased production of ncRNAs may additionally activate sensing platforms in endosomes or cytosol and trigger local inflammation. Another hypothesis could be that syncytin transcripts may align with lncRNA regulatory or R-loop sequences and interfere with transcription of neighboring genes, for which a clear role in obstetric adverse outcomes or SLE has been demonstrated, Figure 2.

This study has several limitations. One is due to its purely computational nature, which indeed requires further confirmation of the results by laboratory experiments on cells and tissues from SLE patients with pregnancy complications. Moreover, this study did not investigate the alignments between human ncRNAs and env genes of other HERV members, such as HERV-F or HERV-K (HML2) proviruses, which may have complementary or opposite functions to syncytins during placentation and embryogenesis [35].

Finally, the wild-type FASTA sequence of syncytin-1 and syncytin-2 genes was used in the present analysis. Therefore, the effects of polymorphic variants, some of which have been associated with the risk of pregnancy complications [86], were not examined with respect to alignment with human ncRNAs.

## 4. Materials and Methods

### 4.1. Identification of Human ncRNA Genes with Complementary Sequences to Syncytin-1 and Syncytin-2 Genes

The FASTA nucleotide sequence of group W endogenous retrovirus, member 1, envelope (ERVW-1) was retrieved from https://www.ncbi.nlm.nih.gov/nuccore/NC_000007.14?report=fasta&from=92468380&to=92477946&strand=true (NCBI reference sequence: NC_000007.14; accessed on 28 December 2022), whereas the FASTA nucleotide sequence of group FRD endogenous retrovirus, member 1, envelope (ERVFRD-1) was retrieved from https://www.ncbi.nlm.nih.gov/nuccore/NC_000006.12?report=fasta&from=11102489&to=11111725&strand=true (NCBI reference sequence: NC_000006.12; accessed on 28 December 2022). In both cases, FASTA sequences were used separately as key input to search for complementary human ncRNA genes in the Ensembl.org library (Human GRCh38.p13) [87]. BLASTN analysis was performed using default settings (normal search sensitivity; maximum number of hits to report: 100; maximum E-value for reported alignment: 10; maximum HSPs per hit: 100; match/mismatch scores: 1.3; gap penalties: opening 2; extension 2).

### 4.2. Analysis of the Molecular Interactions and Biological Function of Human ncRNAs

Human ncRNAs showing alignments with syncytin-1 and syncytin-2 genes and neighboring genes of retrieved ncRNAs were functionally characterized by consulting the following online bioinformatics tools: Ensembl.org [87] for genomic localization and neighboring gene identification; GeneCards database (https://www.genecards.org; accessed on 28 December 2022) [88] for subcellular localization and tissue expression; R-loop Forming Sequence (RLFS) finder [89] for prediction of R-loops in the complementary sequence; RNAct (https://www.rna-society.org/rnainter/home.html; accessed on 28 December 2022) [90] for RNA interactome study. The bioinformatics tool NcPath (http://ncpath.pianlab.cn/#/Home; accessed on 28 December 2022) was used for enrichment analysis of ncRNAs and prediction of KEGG pathways.

### 4.3. Analysis of Polymorphic Variants of Human ncRNAs and Adjacent Coding Genes and Associated Diseases

GeneCards database (https://www.genecards.org; accessed on 28 December 2022) [88] was used to search for associations between polymorphic variants of complementary ncRNAs or adjacent coding genes and human diseases. UniProt Atlas (https://www.uniprot.org; accessed on 28 December 2022) [91] was also queried for biological characterization of proteins encoded by genes adjacent to the ncRNAs of interest.

## 5. Conclusions

This is the first computational study aimed at evaluating a possible epigenetic perturbation in the syncytin-1 and syncytin-2 pathways triggered by the imbalanced expression of ncRNAs. The results of this pivotal analysis suggest that unfavorable pregnancy outcomes may be due to altered crosstalk between lncRNAs and syncytin-1 and syncytin-2 genes or transcripts, ultimately leading to the decreased production of these HERV-derived env proteins during syncytiotrophoblast formation or affecting the expression of other genes critical for placentation or the immune response. However, it is unclear whether these results may be applicable to SLE women who experience pregnancy complications. Further in vitro or ex vivo experiments should be performed to confirm this hypothesis.

## Figures and Tables

**Table 1:** LncRNAs having a complementary sequence to the syncytin-1 gene in a regulatory region, and features of adjacent protein-coding genes (ATF7IP: activating transcription factor 7 interacting protein; BEND4: BEN domain-containing 4; CNR1: cannabinoid receptor 1; CTCF: CCCTC-binding factor; FGF: fibroblast growth factor; GRIN2B: glutamate ionotropic receptor NMDA-type subunit 2B; HERPUD2: homocysteine-inducible, endoplasmic reticulum stress-inducible, ubiquitin-like domain member 2; lncRNA: long noncoding RNA; MARCH: multinucleated neurons, anhydramnios, renal dysplasia, cerebellar hypoplasia and hydranencephaly; PM20D1: peptidase M20 domain-containing 1; RNGTT: RNA guanylyltransferase and 5’-phosphatase; SHISA3: Shisa family member 3; TMPRSS15: transmembrane protease serine 15; Wnt: wingless-related integration site).

Transcript	LncRNA Gene Hit	Type of Regulatory Region	Adjacent Coding Genes	Coded Protein Function	Associated Disease
*ENST00000653218.1* *ENST00000671206.1* *ENST00000355189.7* *ENST00000656413.1* *ENST00000671206.1*	*MIR548XHG*	promoter	*TMPRSS15*	conversion of the pancreatic proenzyme trypsinogen to trypsin	enteropeptidase deficiency; diarrhea; acute pancreatitis; Noonan syndrome 8
*ENST00000669402.1*	*ENSG00000234426*	CTCF	*CNR1*	cannabinoid receptor 1	cannabis abuse; anxiety; chronic pain
			*RNGTT*	bifunctional mRNA-capping	cecum cancer; photokeratitis
*ENST00000605778.1*	*HERPUD2-AS1*	promoter	*HERPUD2*	endoplasmic reticulum unfolded protein response and spermatogenesis	/
			*SEPTIN7*	organization of the actin cytoskeleton, mitosis, cytokinesis and ciliogenesis	amyotrophic neuralgia; brachial plexopathy; extradural neoplasms; MARCH
*ENST00000685812.2*	*ENSG00000289643*	enhancer	*BEND4*	DNA-binding activity	retinitis pigmentosa; colon lymphoma
			*SHISA3*	modulation of both Wnt and FGF signaling pathways	/
*ENST00000605778.1*	*HERPUD2-AS1*	promoter + promoter flank	*HERPUD2* *SEPTIN7*	as above	as above
*ENST00000453774.2* *ENST00000671356.1* *ENST00000665109.1* *ENST00000664286.1* *ENST00000621006.1* *ENST00000658168.1* *ENST00000603129.6* *ENST00000671252.1* *ENST00000627270.2* *ENST00000626601.2* *ENST00000616475.4* *ENST00000626008.2*	*LINC01320*	enhancer	*/*		/
*ENST00000664067.1*	*ENSG00000286619*	promoter + promoter flank + CTCF + enhancer	*PM20D1*	hydrolase and peptidase activity	transient arthritis; Norwegian scabies
*ENST00000538329.1*	*ENSG00000256084*	enhancer	*GRIN2B*	subunit of the NMDA receptor ion channel having agonist binding site for glutamate;	epileptic encephalopathy; intellectual developmental disorder; astigmatism
			*ATF7IP*	regulation of transcription and chromatin formation	liver, testis, and uterus cancer; optic atrophy; alpha thalassemia-intellectual disability syndrome type 1

**Table 2:** LncRNA transcripts complementary to syncytin-1 gene that were predicted to interact with RNA-binding proteins (AEBP2: AE-binding protein 2; CHIC1: cysteine-rich hydrophobic domain-containing protein 1; NISCH: nischarin; SUPT5H: transcription elongation factor SPT5).

Transcript	Gene	Regulatory Sequence	RNA-Binding Protein	Prediction Score
ENST00000413763.1	*ENSG00000226854*	no	NISCH	33.39
ENST00000436786.1	*LINC01239*	no	AEBP2	15.07
ENST00000605778.1	*HERPUD2-AS1*	promoter	SUPT5H	17.65
ENST00000355189.7	*MIR548XHG*	promoter	NISCH	20.06
ENST00000440150.5	*WARS2-AS1*	no	NISCH	30.96
ENST00000444536.1	*LINC00395*	no	NISCH	26.5
ENST00000653218.1	*MIR548XHG*	promoter	NISCH	20.06
ENST00000566193.1	*ENSG00000260197*	no	AEBP2	11.71
ENST00000621006.1	*LINC01320*	enhancer	AEBP2	27.43
ENST00000627270.2	*LINC01320*	enhancer	AEBP2	23.97
ENST00000626601.2	*LINC01320*	enhancer	AEBP2	22.97
ENST00000616475.4	*LINC01320*	enhancer	AEBP2	25.47
ENST00000626008.2	*LINC01320*	enhancer	AEBP2	21.37
ENST00000429137.1	*ENSG00000234426*	no	NISCH	16.79
ENST00000628407.2	*LINC01320*	no	AEBP2	19.44
ENST00000625995.2	*LINC01320*	no	NISCH	23.28
ENST00000433664.1	*LINC00383*	no	AEBP2	30.41
ENST00000413763.1	*ENSG00000226854*	no	NISCH	33.39
ENST00000444770.1	*ENSG00000228566*	no	CHIC1	18.28
ENST00000605778.1	*HERPUD2-AS1*	promoter + promoter flank	SUPT5H	17.65
ENST00000444536.1	*LINC00395*	no	NISCH	26.5
ENST00000623391.1	*ENSG00000280341*	no	NISCH	15.93
ENST00000623391.1	*ENSG00000280341*	no	NISCH	15.93
ENST00000538329.1	*ENSG00000256084*	enhancer	AEBP2	22.08
ENST00000440150.5	*WARS2-AS1*	no	NISCH	30.96
ENST00000621006.1	*LINC01320*	no	AEBP2	27.43
ENST00000436786.1	*LINC01239*	no	AEBP2	15.07

**Table 3:** LncRNAs having a complementary sequence to the syncytin-2 gene in a regulatory region, and features of adjacent protein-coding genes (ARF6: ADP-ribosylation factor 6; BPNT2: 3’(2’), 5’-bisphosphate nucleotidase 2; CTCF: CCCTC-binding factor; CYP8B1: cytochrome P450 family 8 subfamily B member 1; CYTH1: cytohesin-1; DNAH17: dynein axonemal heavy chain 17; EIF1B: eukaryotic translation initiation factor 1b; GLRX5: glutaredoxin-related protein 5; IDDSSBA: intellectual developmental disorder with short stature and behavioral abnormalities; lncRNA: long noncoding RNA; MBL2: mannose-binding lectin 2; MRPS35: mitochondrial 28S ribosomal protein S35; NF-kB: nuclear factor kappa-light-chain-enhancer of activated B cells; NPFFR2: neuropeptide FF receptor 2; REP15: rab15 effector protein; SUDS3: SDS3 homolog, Sin3A corepressor complex component; TCL6: T-cell leukemia/lymphoma 6; ZNF572: zinc finger protein 572; ZNF662: zinc finger protein 662).

Transcript	LncRNA Gene Hit	Type of Regulatory Region	Adjacent Coding Genes	Coded Protein Function	Associated Disease
*ENST00000471537.3*	*ENSG00000273328*	enhancer	*CYP8B1*	cytochrome P450 monooxygenase involved in lipid metabolism and bile acid biosynthesis;	intrahepatic cholestasis of pregnancy; extrahepatic cholestasis; cerebrotindinous xanthomatosis
			*ZNF662*	transcription regulatory activity	/
*ENST00000586185.2*	*SCAT1*	enhancer	*DNAH17*	sperm motility	spermatogenic failure; infertility; pontocerebellar hypoplasia
			*CYTH1*	membrane trafficking, junctional remodeling, and epithelial polarization through regulation of ARF6 activity	IDDSSBA; ankylosing spondylitis; psoriasis
*ENST00000629723.2*	*EIF1B-AS1*	enhancer	*EIF1B*	translation and protein biosynthesis	uveal melanoma; choroid cancer; transposition of the great arteries
*ENST00000538640.2*	*ENSG00000256504*	enhancer	*REP15*	regulation of transferrin receptor recycling from the endocytic recycling compartment	Eiken syndrome; pancreatic adenocarcinoma
			*MRPS35*	mitochondrial ribosomal protein involved in mitochondrial translation and metabolism of proteins	/
*ENST00000657102.1*	*LINC02128*	enhancer	*CYLD*	regulation of inflammation and innate immune response through the control of NF-kB activation	Brooke–Spiegler syndrome; frontotemporal dementia; amyotrophic lateral sclerosis; trichoepithelioma; cylindromatosis; spiradenoma
*ENST00000507156.1*	*ENSG00000248567*	enhancer	*GC*	vitamin-D-binding protein	rickets; osteomalacia; hepatic encephalopathy; blastomycosis; osteoporosis
			*NPFFR2*	neuropeptide receptor interacting with morphine-modulating peptides	postsurgical hypothyroidism; nutmeg liver
*ENST00000654808.1* *ENST00000668157.1*	*LINC02672*	enhancer	*MBL2*	mannose-binding protein C involved in innate immune defense	MBL and complement deficiency; vulvovaginal candidiasis; rheumatic fever; cystic fibrosis
*ENST00000688783.1*	*PURPL*	promoter		/	/
*ENST00000610630.1*	*ENSG00000275409*	enhancer	*SUDS3*	repression of transcription	/
*ENST00000656094.1*	*LINC00964*	promoter + CTCF	*ZNF572*	transcriptional regulation	posterior myocardial infarction
*ENST00000657673.1* *ENST00000659262.1* *ENST00000654139.1* *ENST00000666470.1* *ENST00000660299.1* *ENST00000654721.1* *ENST00000656453.1* *ENST00000667101.1* *ENST00000671217.1* *ENST00000663502.1*	*LINC02318*	enhancer	*GLRX5*	mitochondrial iron–sulfur cluster transfer	sideroblastic anemia; childhood-onset spasticity with hyperglycinemia
			*TCL6*	modulation of the EGFR/AKT pathway at least in placental tissue	lymphoma; leukemia; renal cell carcinoma
*ENST00000661539.1* *ENST00000654770.1* *ENST00000657454.1* *ENST00000661856.1* *ENST00000519241.6* *ENST00000517611.1* *ENST00000519160.5* *ENST00000521132.1* *ENST00000520929.1* *ENST00000655105.1*	*LINC01606*	CTCF	*BPNT2*	Golgi-resident adenosine 3’,5’-bisphosphate 3’-phosphatase with 3’-nucleotidase activity	chondrodysplasia with joint dislocations; ring dermoid of the cornea

**Table 4:** LncRNA transcripts complementary to syncytin-2 gene that were predicted to interact with RNA-binding proteins (AEBP2: AE-binding protein 2; CHIC1: cysteine-rich hydrophobic domain-containing protein 1; DNAJ5B1: DNAJ heat shock protein family (Hsp40) member C5 beta; NISCH: nischarin).

**Transcript**	**Gene**	**Regulatory** **Sequence**	**RNA-Binding Protein**	**Prediction Score**
ENST00000471537.3	*ENSG00000273328*	no	CHIC1	11.96
ENST00000471537.3	*ENSG00000273328*	no	CHIC1	11.96
ENST00000496604.5	*ENSG00000273328*	no	NISCH	18.28
ENST00000471537.3	*ENSG00000273328*	no	CHIC1	11.96
ENST00000444770.1	*ENSG00000228566*	no	CHIC1	18.28
ENST00000471537.3	*ENSG00000273328*	enhancer	CHIC1	11.96
ENST00000426240.5	*LINC02263*	no	AEBP2	33
ENST00000522213.5	*ENSG00000254367*	no	NISCH	20.7
ENST00000626008.2	*ENSG00000256504*	enhancer	AEBP2	16.19
ENST00000438428.1	*LINC01732*	no	DNAJC5B	16.73
ENST00000435023.1	*LINC01732*	no	NISCH	18.9
ENST00000562167.1	*ENSG00000261400*	no	NISCH	14.36
ENST00000507156.1	*ENSG00000248567*	enhancer	CHIC1	24.39
ENST00000456446.1	*ENSG00000226681*	no	AEBP2	21.7
ENST00000556346.1	*LINC02318*	no	NISCH	19.51
ENST00000610630.1	*ENSG00000275409*	enhancer	AEBP2	27.62
ENST00000562167.1	*ENSG00000261400*	no	NISCH	14.36
ENST00000423197.2	*LINC01777*	no	NISCH	26.26
ENST00000635002.1	*LINC01777*	no	NISCH	33.73
ENST00000306533.8	*ENSG00000255689*	no	AEBP2	9.82
ENST00000517611.1	*LINC01606*	CTCF	NISCH	15.33
ENST00000519160.5	*LINC01606*	CTCF	AEBP2	19.36
ENST00000521132.1	*LINC01606*	CTCF	NISCH	16.57
ENST00000520929.1	*LINC01606*	CTCF	NISCH	23.5

**Table 5:** Summary of literature data showing a possible role of lncRNAs aligning with the nucleotide sequence of syncytin-1 and syncytin-2 in the pathogenesis of SLE or pregnancy complications (GWAS: genome-wide association study; LN: lupus nephritis).

**Syncytin-1**	**Aligned lncRNA Gene**	**Potential Contribution to SLE Pathogenesis**	**Potential Contribution to Pregnancy Complications**
	*TP53TG1*	Hypo-expressed in glomerulosclerosis kidney samples according to a molecular signature study of 51 patients with lupus nephritis [55]	Demethylated in female cadmium-exposed placenta according to a genome-wide DNA methylation study of placental tissue from 24 women [45]
	*XACT*	Unknown	Hyper-expressed in both human preimplantation embryos and naive human embryonic stem cells; competes with XIST and prevents X chromosome silencing and functional nullisomy during early human development according to a single-cell RNA-sequencing analysis of more than 100 human embryos [49]; *XACT* loss of heterozygosity potentially affecting the inactivation of the skewed X chromosome and leading to X chromosome instability in human embryonic stem cells revealed by a high-resolution chromosome microarray analysis of 105 human embryos and derived human embryonic stem cells [50]
	*MIR548XHG*	Unknown	Overexpressed in plasma extracellular vesicles from women with endometriosis according to an RNA-sequencing study of 85 patients and 86 controls [51]
	*LINC01239*	Associated with incomplete lupus erythematosus according to a GWAS of 335 patients and 236 controls [56]; Upregulated in morning urine samples from 3 LN patients compared to 3 healthy controls [57]	Dysregulated in patients with epithelial ovarian cancer and endometriosis according to a ChIP-sequencing and ATAC-sequencing analysis of a large cohort of endometriosis and epithelial ovarian cancer patients [52]
	*TTTY14*	Unknown	Hyper-expressed in endometrial samples from infertile women as a phenomenon of male microchimerism according to a transcriptomic profiling study of 60 fertile and infertile participants without endometriosis and 60 fertile and infertile participants with endometriosis [53]
	*LINC01320*	Associated with the inflammatory proximal tubule histologic subtype observed in kidney samples from patients with glomerulonephritis, including one case of LN, according to a single-cell RNA-sequencing study [58]	Upregulated in the endometrium during the implantation window according to an RNA-sequencing analysis of 30 fertile women [48]; Dysregulated in syncytiotrophoblasts, invasive cytotrophoblasts, and endovascular cytotrophoblasts isolated from placental tissue of 4 women with severe preeclampsia and 4 women with uninfected preterm birth according to a global transcriptional profiling study [46]
**Syncytin-2**	*XACT*	Unknown	As above
	*LINC00320*	Unknown	Upregulated in spontaneous preterm placenta from 20 women compared to spontaneous term placenta from 20 control subjects according to a transcriptomic RNA-sequencing analysis [47]
	*TP53TG1*	As above	As above

**Table 6:** Results of studies reporting associations between protein-coding genes adjacent to lncRNA genes with hits in the syncytin-1 and syncytin-2 nucleotide sequence and SLE or pregnancy adverse outcomes (ERα: estrogen receptor α; GWAS: genome-wide association study; ISN/RPS: International Society of Nephrology/Renal Pathology Society; LN: lupus nephritis; qPCR: quantitative polymerase chain reaction; qRT-PCR: quantitative real time-polymerase chain reaction; SNPs: single nucleotide polymorphisms).

**Syncytin-1**	**Aligned lncRNA Gene**	**Adjacent Protein-coding Gene**	**Data Supporting an Association with SLE**	**Data Supporting an Association with Pregnancy Adverse Outcomes**
	*ENSG00000234426*	*CNR1*	Unknown	Increased risk of preeclampsia according to a case-control study genotyping 115 preeclamptic women and 145 healthy pregnant controls [65]
	*ENSG00000256084*	*GRIN2B*	Included among the new SLE genes according to an OASIS analysis of 6077 subjects [67]	Hypermetilated in leukocytes of preeclamptic women compared with normotensive pregnant women according to a genome-wide methylation profiling study of 28 participants [66]
**Syncytin-2**	*LINC02128*	*CYLD*	Overexpressed in kidney samples from 4 patients with class II and class IV LN according to ISN/RPS 2003 criteria [76]	Candidate predictor for preterm birth according to an mRNA-sequencing analysis of 88 Korean preterm births and 118 control subjects [68]
	*ENSG00000248567*	*GC*	Unknown	SNPs associated with risk of preterm birth according to a prospective cohort study genotyping 3465 pregnant women, of whom 202 were preterm [69]
	*LINC02672*	*MBL2*	Association of A/B and A/O polymorphisms with SLE susceptibility, and protective effect of allele H according to a meta-analysis of 7194 SLE patients and 7401 healthy controls [77]; Association between the *MBL2* O allele and low MBL producer genotypes and increased SLE risk according to a genotyping study of 34 Brazilian SLE patients and 101 controls [78]	Higher frequency of codon 52 polymorphism in preterm birth cases compared with term controls and association of *MBL2* O/O genotype with risk of preterm birth according to a genotyping study of 204 DNA blood samples [70]; Association between *MBL2* genotypes leading to MBL deficiency and recurrent late pregnancy loss independent of LAC positivity according to a genotyping study of 75 patients and 104 controls [72]
	*ENSG00000273328*	*ZNF572*	Unknown	Upregulated in amniotic fluid supernatant samples from 21 preterm birth patients compared to term birth controls according to a sequencing and qPCR study [71]
	*ENSG00000273328*	*CYP8B1*	Unknown	Undergoes ERα-induced downregulation in mice, leading to impaired bile acid biosynthesis and potential risk of intrahepatic cholestasis in pregnancy [73]
	*LINC02318*	*TCL6*	Unknown	Overexpressed in 42 placental tissues from women with preeclampsia compared with controls and hypo-expressed in preeclamptic pregnancies with lower urine protein levels, normal blood pressure, and higher newborn weight according to a qRT-PCR study [74];Overexpressed in placental tissue in threatened abortion pregnancy compared with normal pregnancy and in spontaneous abortion pregnancy compared with induced abortion pregnancy according to a qRT-PCR study of 30 women with spontaneous abortion, 30 women with induced abortion, and 30 control subjects with normal pregnancy [75]
	*ENSG00000248567*	*NPFFR2*	Unknown	Hyper-expressed in placental tissue during the first trimester and in placental samples from preeclamptic women and indirectly associated with the expression of syncytin-1 and syncytin-2 in human cytotrophoblast cells [36]

## Data Availability

Not applicable.

## Supplementary Materials

The following supporting information can be downloaded at: https://www.mdpi.com/article/10.3390/ijms24032259/s1.
