# Supplementary material for: Interaction between Long Noncoding RNAs and Syncytin-1/Syncytin-2 Genes and Transcripts: How Noncoding RNAs May Affect Pregnancy in Patients with Systemic Lupus Erythematosus
Source: Int J Mol Sci. 2023 Jan 23;24(3):2259. doi: 10.3390/ijms24032259 (PMC9917164; doi:10.3390/ijms24032259)
Supplement: Supplementary file 1 [file ijms-24-02259-s001.zip › Table S1.pdf]

TRANSCRIPT	GENOMIC LOCATION	GENE HIT	LENGTH	SCORE	%ID	EXPRESSION	EXPRESSED IN PLACENTA	SUBCELLULAR LOCALISATION	ASSOCIATED GENETIC DISEASE
**ENST00000658227.1**	6:166314937-166316243	*ENSG00000287189*	1312	1650	90.9	right uterine tube, olfactory segment of nasal mucosa, hypothalamus and other tissues	/	/	
**ENST00000670000.1**	1:119180392-119181234	*WARS2-AS1*	852	931	88.8	ubiquitous	NO	/	Parkinsonism-Dystonia 3, childhood-onset
**ENST00000413763.1**	X:75903868-75904856	*ENSG00000226854*	1012	765	83.7	nervous system, gastrointestinal tract, glands, ovary, prostate	NO	/	
**ENST00000674361.1**	X:113897095-113897617	*XACT*	524	700	91.9	pancreas, colon, testis	NO	nucleolus	
**ENST00000670000.1**	1:119181237-119181954	*WARS2-AS1*	718	672	86.6	ubiquitous	NO	/	Parkinsonism-Dystonia 3, childhood-onset
**ENST00000670000.1**	1:119182141-119182963	*WARS2-AS1*	865	654	83.5	ubiquitous	NO	/	Parkinsonism-Dystonia 3, childhood-onset
**ENST00000440150.5**	1:119182141-119182963	*WARS2-AS1*	865	654	83.5	ubiquitous	NO	/	Parkinsonism-Dystonia 3, childhood-onset
**ENST00000653218.1**	21:18759799-18760297	*MIR548XHG*	499	615	90.5	brain, glands, intestine, skin, testis	NO	/	
**ENST00000671206.1**	21:18759799-18760297	*MIR548XHG*	499	615	90.5	brain, glands, intestine, skin, testis	NO	/	
**ENST00000685812.2**	4:42285839-42286367	*ENSG00000289643*	529	613	89.4	/	/	/	
**ENST00000657223.1**	13:69221874-69222446	*LINC00383*	575	577	87.3	gonad, testis, thyroid, brain	NO	/	Hepatocellular carcinoma
**ENST00000669402.1**	6:88420946-88421373	*ENSG00000234426*	428	569	91.8	overexpressed in whole blood	NO	/	/
**ENST00000436786.1**	9:22823297-22823817	*LINC01239*	523	569	88.5	overexpressed in adipose, subcutaneous tissue and breast	NO	/	Fanconi anemia complementation group I
**ENST00000671139.1**	2:112039378-112039935	*ENSG00000286904*	559	544	86.5	colonic epithelium, right adrenal gland cortex, sural nerve and other tissues	/	/	/
**ENST00000454367.2**	2:58275640-58276157	*LINC01795*	526	502	87	testis, brain, intestine, ovary, blood	NO	/	/

**ENST00000655335.1**	2:58275640-58276157	*LINC01795*	526	502	87	testis, brain, intestine, ovary, blood	NO	/	/
**ENST00000605778.1**	7:35696139-35696567	*HERPUD2-AS1*	432	490	88.8	ubiquitous	NO	/	/
**ENST00000670606.1**	2:75872035-75872492	*ENSG00000287474*	459	486	88.4	gonad, testis, pancreas and other tissues	/	/	/
**ENST00000674361.1**	X:113897542-113897869	*XACT*	328	480	93.5	pancreas, colon, testis	NO	nucleolus	/
**ENST00000657054.1**	12:126449360-126449808	*LINC02347*	457	461	87.7	ubiquitous	NO	/	/
**ENST00000661190.1**	12:126449360-126449808	*LINC02347*	457	461	87.7	ubiquitous	NO	/	/
**ENST00000654221.1**	12:126449360-126449808	*LINC02347*	457	461	87.7	ubiquitous	NO	/	/
**ENST00000663153.1**	12:126449360-126449808	*LINC02347*	457	461	87.7	ubiquitous	NO	/	/
**ENST00000653244.1**	12:126449360-126449808	*LINC02347*	457	461	87.7	ubiquitous	NO	/	/
**ENST00000649476.1**	18:57735177-57735590	*ENSG00000267787*	415	453	88.9	colonic epithelium, granulocytes, monocytes and other tissues	/	/	/
**ENST00000588925.5**	18:57735177-57735590	*ENSG00000267787*	415	453	88.9	colonic epithelium, granulocytes, monocytes and other tissues	/	/	/
**ENST00000355189.7**	21:18752749-18759774	*MIR548XHG*	369	453	90.5	brain, glands, intestine, skin, testis	NO	/	
**ENST00000685812.2**	4:42292169-42292599	*ENSG00000289643*	431	417	87.2	/	/	/	/
**ENST00000681309.1**	3:70617120-70617434	*ENSG00000288679*	327	378	89.2	/	/	/	/
**ENST00000414582.1**	21:18752749-18753028	*MIR548XHG*	280	356	.	brain, glands, intestine, skin, testis	NO	/	
**ENST00000664906.1**	2:34732453-34732768	*ENSG00000232153*	316	340	88.6	adipose tissue, kidney, pancreas, testis	NO	/	/
**ENST00000643361.1**	1:68698450-68864146	*ENSG00000285407*	324	330	87.9	ventricular zone, nucleus accumbens, ganglionic eminence and other tissues	/	/	/

**ENST00000530569.5**	11:22317752-22318037	*SLC17A6-DT*	286	289	87.7	brain, muscle, gastrointestinal, glands, testis	NO	/	/
**ENST00000605778.1**	7:35696697-35697010	*HERPUD2-AS1*	314	281	86.3	ubiquitous	NO	/	/
**ENST00000586729.1**	18:57735400-57735590	*ENSG00000267787*	191	259	92.1	colonic epithelium, granulocyte, monocyte and other tissues	/	/	/
**ENST00000656413.1**	21:18759799-18760027	*MIR548XHG*	229	255	89	brain, glands, intestine, skin, testis	NO	/	/
**ENST00000681309.1**	3:70617408-70617661	*ENSG00000288679*	255	241	87	/	/	/	/
**ENST00000695562.1**	13:23527872-23528172	*ENSG00000289688*	301	190	83	/	/	/	/
**ENST00000655335.1**	2:58275536-58275678	*LINC01795*	144	186	91.6	testis, brain, intestine, ovary, blood, kidney	NO	/	/
**ENST00000657054.1**	12:126449766-126449896	*LINC02347*	132	180	92.4	testis, brain, pancreas, intestine, breast, ovary, spleen, blood, glands	NO	/	/
**ENST00000661190.1**	12:126449766-126449896	*LINC02347*	132	180	92.4	testis, brain, pancreas, intestine, breast, ovary, spleen, blood, glands	NO	/	/
**ENST00000654221.1**	12:126449766-126449896	*LINC02347*	132	180	92.4	testis, brain, pancreas, intestine, breast, ovary, spleen, blood, glands	NO	/	/
**ENST00000663153.1**	12:126449766-126449896	*LINC02347*	132	180	92.4	testis, brain, pancreas, intestine, breast, ovary, spleen, blood, glands	NO	/	/
**ENST00000653244.1**	12:126449766-126449896	*LINC02347*	132	180	92.4	testis, brain, pancreas, intestine, breast, ovary, spleen, blood, glands	NO	/	/

**ENST00000670000.1**	1:119182983-119183225	*WARS2-AS1*	246	178	84.1	ubiquitous	NO	/	Parkinsonism-Dystonia 3, childhood-onset
**ENST00000440150.5**	1:119182983-119183225	*WARS2-AS1*	246	178	84.1	ubiquitous	NO	/	Parkinsonism-Dystonia 3, childhood-onset
**ENST00000667450.1**	Y:19040249-19040543	*TTTY14*	295	178	82.3	ubiquitous	**YES**	nucleus	/
**ENST00000658227.1**	6:166316268-166316382	*ENSG00000287189*	115	164	93	right uterine tube, olfactory segment of nasal mucosa, hypothalamus and other tissues	NO	/	/
**ENST00000648574.1**	13:69222324-69222446	*LINC00383*	124	164	91.9	brain, testis, prostate	NO	/	Hepatocellular carcinoma
**ENST00000444536.1**	13:63687404-63687509	*LINC00395*	106	154	93.3	mainly testis	NO	/	/
**ENST00000653218.1**	21:18760222-18760312	*MIR548XHG*	91	148	95.6	brain, glands, intestine, skin, testis	NO	/	/
**ENST00000671206.1**	21:18760222-18760312	*MIR548XHG*	91	148	95.6	brain, glands, intestine, skin, testis	NO	/	/
**ENST00000702094.1**	7:87329938-87330060	*TP53TG1*	123	140	89.4	ubiquitous	**YES**	nucleus, cytosol, mitochondrion, cytoskeleton, extracellular, plasma membrane	Glioma, lung and colorectal cancer
**ENST00000687819.1**	7:87329938-87330060	*TP53TG1*	123	140	89.4	ubiquitous	**YES**	nucleus, cytosol, mitochondrion, cytoskeleton, extracellular, plasma membrane	Glioma, lung and colorectal cancer
**ENST00000686297.1**	7:87329938-87330060	*TP53TG1*	123	140	89.4	ubiquitous	**YES**	nucleus, cytosol, mitochondrion, cytoskeleton, extracellular, plasma membrane	Glioma, lung and colorectal cancer
**ENST00000359941.11**	7:87329938-87330060	*TP53TG1*	123	140	89.4	ubiquitous	**YES**	nucleus, cytosol, mitochondrion, cytoskeleton, extracellular, plasma membrane	Glioma, lung and colorectal cancer
**ENST00000566193.1**	Y:19694443-19694603	*ENSG00000260197*	162	136	85.8	ubiquitous	NO	/	/
**ENST00000453774.2**	2:34732453-34737321	*LINC01320*	106	130	90.5	kidney, testis, pancreas, uterus,	NO	/	/

						brain, heart, intestine			
**ENST00000671356.1**	2:34732453-34737321	*LINC01320*	106	130	90.5	kidney, testis, pancreas, uterus, brain, heart, intestine	NO	/	/
**ENST00000665109.1**	2:34732453-34737321	*LINC01320*	106	130	90.5	kidney, testis, pancreas, uterus, brain, heart, intestine	NO	/	/
**ENST00000664286.1**	2:34732453-34737321	*LINC01320*	106	130	90.5	kidney, testis, pancreas, uterus, brain, heart, intestine	NO	/	/
**ENST00000621006.1**	2:34732453-34737321	*LINC01320*	106	130	90.5	kidney, testis, pancreas, uterus, brain, heart, intestine	NO	/	/
**ENST00000658168.1**	2:34732453-34737321	*LINC01320*	106	130	90.5	kidney, testis, pancreas, uterus, brain, heart, intestine	NO	/	/
**ENST00000603129.6**	2:34732453-34737321	*LINC01320*	106	130	90.5	kidney, testis, pancreas, uterus, brain, heart, intestine	NO	/	/
**ENST00000671252.1**	2:34732453-34737321	*LINC01320*	106	130	90.5	kidney, testis, pancreas, uterus, brain, heart, intestine	NO	/	/
**ENST00000627270.2**	2:34732453-34737321	*LINC01320*	106	130	90.5	kidney, testis, pancreas, uterus, brain, heart, intestine	NO	/	/
**ENST00000626601.2**	2:34732453-34737321	*LINC01320*	106	130	90.5	kidney, testis, pancreas, uterus, brain, heart, intestine	NO	/	/
**ENST00000616475.4**	2:34732453-34737321	*LINC01320*	106	130	90.5	kidney, testis, pancreas, uterus, brain, heart, intestine	NO	/	/
**ENST00000626008.2**	2:34732453-34737321	*LINC01320*	106	130	90.5	kidney, testis, pancreas, uterus,	NO	/	/

						brain, heart, intestine			
**ENST00000429137.1**	6:88420946-88421031	*ENSG00000234426*	86	130	94.1	ubiquitous	NO	/	/
**ENST00000628407.2**	2:34732453-34732557	*LINC01320*	105	128	90.4	kidney, testis, pancreas, uterus, brain, heart, intestine	NO	/	/
**ENST00000625995.2**	2:34732453-34732557	*LINC01320*	105	128	90.4	kidney, testis, pancreas, uterus, brain, heart, intestine	NO	/	/
**ENST00000433664.1**	13:69222349-69222446	*LINC00383*	99	123	90.9	right uterine tube, olfactory segment of nasal mucosa, hypothalamus and other tissues	/	/	/
**ENST00000413763.1**	X:75903105-75903175	*ENSG00000226854*	72	109	94.4	brain, gonads, glands, intestine	NO	/	/
**ENST00000695562.1**	13:23527417-23527535	*ENSG00000289688*	119	109	86.5	/	/	/	/
**ENST00000661265.1**	3:70617766-70617836	*ENSG00000287669*	71	109	94.3	/	/	/	/
**ENST00000695562.1**	13:23528606-23528775	*ENSG00000289688*	170	107	82.9	/	/	/	/
**ENST00000690980.1**	7:87329938-87330031	*TP53TG1*	94	107	89.3	ubiquitous	**YES**	nucleus, cytosol, mitochondrion, cytoskeleton, extracellular, plasma membrane	Glioma, lung and colorectal cancer
**ENST00000444770.1**	10:63664664-63664770	*ENSG00000228566*	107	107	86.9	ubiquitous	NO	/	/
**ENST00000605778.1**	7:35696529-35696701	*HERPUD2-AS1*	181	105	82.3	ubiquitous	NO	/	/
**ENST00000657223.1**	13:69221866-69221946	*LINC00383*	82	103	91.4	right uterine tube, olfactory segment of nasal mucosa, hypothalamus and other tissues	/	/	/
**ENST00000695562.1**	13:23525374-23525565	*ENSG00000289688*	192	97.4	81.7	/	/	/	/
**ENST00000444536.1**	13:63687493-63714355	*LINC00395*	67	93.4	92.5	mainly testis	NO	/	/

**ENST00000623391.1**	22:15287138-15287367	*ENSG00000280341*	232	93.4	79.3	pancreas, cortical plate, blood and other tissues	/	/	/
**ENST00000623391.1**	22:15286984-15287089	*ENSG00000280341*	106	91.4	85.8	pancreas, cortical plate, blood and other tissues	/	/	/
**ENST00000664067.1**	1:205858319-205868833	*ENSG00000286619*	66	91.4	92.4	esophagus, brain, pancreas and other tissues	/	/	/
**ENST00000538329.1**	12:14220974-14221112	*ENSG00000256084*	140	77.6	82.1	ubiquitous	NO	/	/
**ENST00000660850.1**	2:58276096-58276157	*LINC01795*	62	75.6	90.3	testis, brain, intestine, ovary, blood, kidney	NO	/	/
**ENST00000670000.1**	1:119183198-119183268	*WARS2-AS1*	81	73.6	83.9	ubiquitous	NO		Parkinsonism-Dystonia 3, childhood-onset
**ENST00000440150.5**	1:119183198-119183268	*WARS2-AS1*	81	73.6	83.9	ubiquitous	NO		Parkinsonism-Dystonia 3, childhood-onset
**ENST00000685812.2**	4:42292572-42292644	*ENSG00000289643*	83	69.7	83.1	/	/	/	/
**ENST00000454367.2**	2:58275640-58275678	*LINC01795*	39	69.7	97.4	testis, brain, intestine, ovary, blood, kidney	NO	/	/
**ENST00000664906.1**	2:34732421-34732466	*ENSG00000232153*	47	67.7	93.6	testis, adipocytes, kidney	NO	/	/
**ENST00000621006.1**	2:34732421-34732466	*LINC01320*	47	67.7	93.6	kidney, testis, pancreas, uterus, brain, heart, intestine	NO	/	/
**ENST00000702818.1**	11:110328463-110328521	*ENSG00000290078*	59	67.7	89.8	/	/	/	/
**ENST00000685812.2**	4:42292109-42292172	*ENSG00000289643*	64	63.8	87.5	/	/	/	/
**ENST00000436786.1**	9:22823252-22823291	*LINC01239*	40	63.8	95	ubiquitous, overexpressed in adipose, subcutaneous and mammary tissue	NO	/	/
**ENST00000657054.1**	12:126449284-126449319	*LINC02347*	36	63.8	97.2	testis, brain, pancreas, intestine, breast, ovary, spleen, blood, glands	NO	/	/

**ENST00000661190.1**	12:126449284-126449319	*LINC02347*	36	63.8	97.2	testis, brain, pancreas, intestine, breast, ovary, spleen, blood, glands	NO	/	/
**ENST00000654221.1**	12:126449284-126449319	*LINC02347*	36	63.8	97.2	testis, brain, pancreas, intestine, breast, ovary, spleen, blood, glands	NO	/	/
