# Supplementary material for: Interaction between Long Noncoding RNAs and Syncytin-1/Syncytin-2 Genes and Transcripts: How Noncoding RNAs May Affect Pregnancy in Patients with Systemic Lupus Erythematosus
Source: Int J Mol Sci. 2023 Jan 23;24(3):2259. doi: 10.3390/ijms24032259 (PMC9917164; doi:10.3390/ijms24032259)
Supplement: Supplementary file 1 [file ijms-24-02259-s001.zip › Table S2.pdf]

TRANSCRIPT	GENOMIC LOCATION	GENE HIT	LENGTH	SCORE	%ID	EXPRESSION	EXPRESSED IN PLACENTA	SUBCELLULAR LOCALIZATION	ASSOCIATED DISEASE
**ENST00000663151.1**	2:828293-829246	*LINC01115*	985	346	79.2	ubiquitous	NO	/	/
**ENST00000659656.1**	2:828293-829246	*LINC01115*	985	346	79.2	ubiquitous	NO	/	/
**ENST00000649319.1**	7:153396140-153396818	*ENSG00000285524*	702	265	79.7	muscle, heart	NO	/	/
**ENST00000471537.3**	3:42897057-42897334	*ENSG00000273328*	281	233	85.7	liver, placenta, nerves	**YES**	/	/
**ENST00000471537.3**	3:42897475-42898063	*ENSG00000273328*	604	233	79.9	liver, placenta, nerves	**YES**	/	/
**ENST00000659515.1**	8:68307367-68307715	*C8orf34-AS1*	352	233	83.5	ubiquitous	NO	/	/
**ENST00000496604.5**	3:42897057-42897324	*ENSG00000273328*	271	213	85.2	liver, placenta, nerves	**YES**	/	/
**ENST00000471537.3**	3:42898080-42898361	*ENSG00000273328*	286	210	84.6	liver, placenta, nerves	**YES**	/	/
**ENST00000683405.1**	3:46001123-46001283	*ENSG00000288717*	161	184	89.4	/	/	/	/
**ENST00000627700.2**	X:98864789-98864966	*ENSG00000281566*	180	164	87.2	monocytes, muscles, lymph nodes	/	/	/
**ENST00000626665.3**	X:98864789-98864966	*ENSG00000281566*	180	164	87.2	monocytes, muscles, lymph nodes	/	/	/
**ENST00000654670.1**	4:117360610-117360807	*LINC02263*	199	154	84.9	brain, testis	NO	/	/
**ENST00000668339.1**	4:117360610-117360807	*LINC02263*	199	154	84.9	brain, testis	NO	/	/
**ENST00000444770.1**	10:63990452-63990568	*ENSG00000228566*	117	152	91.4	monocytes, muscles, lymph nodes	NO	/	/
**ENST00000695939.1**	2:216886423-216886752	*DIRC3-AS1*	333	150	81.0	ubiquitous	NO	/	/
**ENST00000674361.1**	X:113893884-113894222	*XACT*	347	150	80.6	pancreas, colon, testis	NO	nucleolus	/
**ENST00000666918.1**	4:117360650-117360807	*LINC02263*	159	146	86.7	brain, testis	NO	/	/
**ENST00000654527.1**	4:117360650-117360807	*LINC02263*	159	146	86.7	brain, testis	NO	/	/
**ENST00000655906.1**	4:117360650-117360807	*LINC02263*	159	146	86.7	brain, testis	NO	/	/
**ENST00000656442.1**	4:117360650-117360807	*LINC02263*	159	146	86.7	brain, testis	NO	/	/
**ENST00000420701.6**	4:117360650-117360807	*LINC02263*	159	146	86.7	brain, testis	NO	/	/
**ENST00000471537.3**	3:42898974-42899153	*ENSG00000273328*	183	119	83.0	liver, placenta, nerves	**YES**	/	/
**ENST00000595201.2**	19:48214214-48214420	*ENSG00000268583*	207	117	82.1	mostly whole blood	NO	/	/
**ENST00000426240.5**	4:117360666-117360807	*LINC02263*	143	115	85.3	brain, testis	NO	/	/
**ENST00000586185.2**	17:78618599-78618799	*SCAT1*	203	115	82.2	bone marrow, nerves, tendons	NO	/	Esophageal cancer
**ENST00000663810.1**	1:224608766-224608878	*CNIH3-AS2*	113	105	86.7	endometrium, brain	NO	/	/
**ENST00000437416.3**	1:224608766-224608878	*CNIH3-AS2*	113	105	86.7	endometrium, brain	NO	/	/
**ENST00000629723.2**	3:40289892-40290229	*EIF1B-AS1*	343	99.3	78.7	gonads, endothelium, colon	/	/	/
**ENST00000668649.1**	14:95573600-95573834	*LINC02318*	237	97.4	80.5	nerves, testis	NO	/	/
**ENST00000668165.1**	14:95573600-95573834	*LINC02318*	237	97.4	80.5	nerves, testis	NO	/	/
**ENST00000656081.1**	5:1349805-1349999	*ENSG00000286388*	200	95.4	81.0	brain, heart	/	/	/
**ENST00000522213.5**	8:8782246-8782432	*ENSG00000254367*	191	95.4	81.6	overexpressed in testis and spleen	NO	/	/
**ENST00000643688.1**	CHR_HG76_PATCH:11174294-11174480	*ENSG00000284943*	191	95.4	81.6	/	/	/	/
**ENST00000653972.1**	21:23423217-23423347	*D21S2088E*	132	93.4	84.0	brain, testis	NO	/	/

**ENST00000538640.2**	12:27708128-27708249	*ENSG00000256504*	123	89.5	84.5	ubiquitous	NO	/	/
**ENST00000438428.1**	1:181182073-181182181	*LINC01732*	112	87.5	85.7	vessels, respiratory epithelium	NO	/	/
**ENST00000435023.1**	1:181182073-181182181	*LINC01732*	112	87.5	85.7	vessels, respiratory epithelium	NO	/	/
**ENST00000657102.1**	16:50887400-50893661	*LINC02128*	79	83.5	88.6	liver, myometrium	NO	/	/
**ENST00000664253.1**	5:60701691-60701908	*ENSG00000287434*	224	79.6	78.5	nerves, bone marrow	/	/	/
**ENST00000663810.1**	1:224608908-224609146	*CNIH3-AS2*	246	75.6	79.2	endometrium, brain	NO	/	/
**ENST00000437416.3**	1:224608908-224609146	*CNIH3-AS2*	246	75.6	79.2	endometrium, brain	NO	/	/
**ENST00000667398.1**	19:31588381-31588426	*THEG5*	46	75.6	95.6	mostly testis	/	/	/
**ENST00000656632.1**	19:31588381-31588426	*THEG5*	46	75.6	95.6	mostly testis	/	/	/
**ENST00000562167.1**	19:31588381-31588426	*ENSG00000261400*	46	75.6	95.6	gonads, brain	/	/	/
**ENST00000507156.1**	4:71821376-71821491	*ENSG00000248567*	116	71.7	82.7	blood, brain, testis	NO	/	/
**ENST00000670475.1**	13:63437551-63437663	*ENSG00000287996*	114	71.7	83.3	mostly brain, glands	/	/	/
**ENST00000654836.1**	21:20777519-20777617	*LINC00320*	99	69.7	83.8	mostly brain	NO	nucleus, peroxisome	Malignant glioma
**ENST00000654808.1**	10:52975808-53022889	*LINC02672*	73	69.7	87.6	mostly uterus and glands	NO	/	/
**ENST00000668157.1**	10:52975808-53022889	*LINC02672*	73	69.7	87.6	mostly uterus and glands	NO	/	/
**ENST00000456446.1**	2:181425536-181425696	*ENSG00000226681*	165	67.7	80.6	testis	NO	/	/
**ENST00000688783.1**	5:27476261-27476353	*PURPL*	93	65.7	83.8	endometrium, colon	NO	nucleus	Colon cancer, congenital myasthenia, thoracic aortic aneurysm, retinitis pigmentosa
**ENST00000654800.1**	7:10085338-10085398	*ENSG00000287409*	61	65.7	88.5	nerves, endometrium	/	/	/
**ENST00000626665.3**	X:98865293-98865376	*ENSG00000281566*	85	63.8	84.7	monocytes, lymph node	/	/	/
**ENST00000656094.1**	8:124962349-124962469	*LINC00964*	121	59.8	80.1	testis and prostate	NO	/	/
**ENST00000657673.1**	14:95573600-95577429	*LINC02318*	86	59.8	83.7	nerves, testis	NO	/	/
**ENST00000659262.1**	14:95573600-95577429	*LINC02318*	86	59.8	83.7	nerves, testis	NO	/	/
**ENST00000556346.1**	14:95573600-95573684	*LINC02318*	85	57.8	83.5	nerves, testis	NO	/	/
**ENST00000663151.1**	2:828107-828146	*LINC01115*	40	55.9	92.5	pituitary gland	NO	/	/
**ENST00000659656.1**	2:828107-828146	*LINC01115*	40	55.9	92.5	pituitary gland	NO	/	/
**ENST00000610630.1**	12:118430186-118430264	*ENSG00000275409*	79	55.9	83.5	brain	NO	/	/
**ENST00000659493.1**	18:58808473-58808516	*LINC01926*	44	55.9	90.9	prostate, bone marrow, lymph node	NO	/	/
**ENST00000654139.1**	14:95573600-95577429	*LINC02318*	84	55.9	83.3	nerves, testis	NO	/	/
**ENST00000666470.1**	14:95573600-95577429	*LINC02318*	84	55.9	83.3	nerves, testis	NO	/	/
**ENST00000660299.1**	14:95573600-95577429	*LINC02318*	84	55.9	83.3	nerves, testis	NO	/	/
**ENST00000654721.1**	14:95573600-95577429	*LINC02318*	84	55.9	83.3	nerves, testis	NO	/	/
**ENST00000656453.1**	14:95573600-95577429	*LINC02318*	84	55.9	83.3	nerves, testis	NO	/	/
**ENST00000667101.1**	14:95573600-95577429	*LINC02318*	84	55.9	83.3	nerves, testis	NO	/	/
**ENST00000671217.1**	14:95573600-95577429	*LINC02318*	84	55.9	83.3	nerves, testis	NO	/	/
**ENST00000663502.1**	14:95573600-95577429	*LINC02318*	84	55.9	83.3	nerves, testis	NO	/	/
**ENST00000667398.1**	19:31588234-31588336	*THEG5*	104	53.9	81.7	mostly testis	/	/	/

**ENST00000656632.1**	19:31588234-31588336	*THEG5*	104	53.9	81.7	mostly testis	/	/	/
**ENST00000562167.1**	19:31588234-31588336	*ENSG00000261400*	104	53.9	81.7	gonads	/	/	/
**ENST00000670434.1**	1:4412146-4412244	*LINC01777*	100	53.9	82.0	bone marrow, nerves, testis	NO	/	/
**ENST00000668086.1**	1:4412146-4412244	*LINC01777*	100	53.9	82.0	bone marrow, nerves, testis	NO	/	/
**ENST00000659315.1**	1:4412146-4412244	*LINC01777*	100	53.9	82.0	bone marrow, nerves, testis	NO	/	/
**ENST00000633821.2**	1:4412146-4412244	*LINC01777*	100	53.9	82.0	bone marrow, nerves, testis	NO	/	/
**ENST00000665284.1**	1:4412146-4412244	*LINC01777*	100	53.9	82.0	bone marrow, nerves, testis	NO	/	/
**ENST00000671656.1**	1:4412146-4412244	*LINC01777*	100	53.9	82.0	bone marrow, nerves, testis	NO	/	/
**ENST00000663417.1**	1:4412146-4412244	*LINC01777*	100	53.9	82.0	bone marrow, nerves, testis	NO	/	/
**ENST00000669931.1**	1:4412146-4412244	*LINC01777*	100	53.9	82.0	bone marrow, nerves, testis	NO	/	/
**ENST00000635312.2**	1:4412146-4412244	*LINC01777*	100	53.9	82.0	bone marrow, nerves, testis	NO	/	/
**ENST00000423197.2**	1:4412146-4412244	*LINC01777*	100	53.9	82.0	bone marrow, nerves, testis	NO	/	/
**ENST00000635002.1**	1:4412146-4412244	*LINC01777*	100	53.9	82.0	bone marrow, nerves, testis	NO	/	/
**ENST00000666295.1**	14:95573600-95573654	*LINC02318*	55	53.9	87.2	nerves, testis	NO	/	/
**ENST00000668634.1**	14:95573600-95573654	*LINC02318*	55	53.9	87.2	nerves, testis	NO	/	/
**ENST00000664519.1**	X:142739904-142739946	*ENSG00000288098*	43	53.9	90.6	testis	/	/	/
**ENST00000663739.1**	13:82778504-82778558	*ENSG00000286385*	56	53.9	87.5	pancreas, nerves, liver	/	/	/
**ENST00000689870.1**	11:115582465-115582507	*ENSG00000255689*	43	51.9	90.6	muscle, nerves	NO	/	/
**ENST00000306533.8**	11:115582465-115582507	*ENSG00000255689*	43	51.9	90.6	muscle, nerves	NO	/	/
**ENST00000661943.3**	7:87323719-87323756	*TP53TG1*	38	51.9	92.1	ubiquitous	**YES**	nucleus, mitochondrion, cytoskeleton	Lung and colorectal cancer
**ENST00000661539.1**	8:57219421-57219482	*LINC01606*	63	51.9	85.7	testis	NO	/	/
**ENST00000654770.1**	8:57219421-57219482	*LINC01606*	63	51.9	85.7	testis	NO	/	/
**ENST00000657454.1**	8:57219421-57219482	*LINC01606*	63	51.9	85.7	testis	NO	/	/
**ENST00000661856.1**	8:57219421-57219482	*LINC01606*	63	51.9	85.7	testis	NO	/	/
**ENST00000519241.6**	8:57219421-57219482	*LINC01606*	63	51.9	85.7	testis	NO	/	/
**ENST00000517611.1**	8:57219421-57219482	*LINC01606*	63	51.9	85.7	testis	NO	/	/
**ENST00000519160.5**	8:57219421-57219482	*LINC01606*	63	51.9	85.7	testis	NO	/	/
**ENST00000521132.1**	8:57219421-57219482	*LINC01606*	63	51.9	85.7	testis	NO	/	/
**ENST00000520929.1**	8:57219421-57219482	*LINC01606*	63	51.9	85.7	testis	NO	/	/
**ENST00000655105.1**	8:57219421-57219482	*LINC01606*	63	51.9	85.7	testis	NO	/	/
